# Viral predation pressure on coral reefs

**DOI:** 10.1186/s12915-023-01571-9

**Published:** 2023-04-11

**Authors:** Cynthia B. Silveira, Antoni Luque, Andreas F. Haas, Ty N. F. Roach, Emma E. George, Ben Knowles, Mark Little, Christopher J. Sullivan, Natascha S. Varona, Linda Wegley Kelly, Russel Brainard, Forest Rohwer, Barbara Bailey

**Affiliations:** 1 Department of Biology, University of Miami, Coral Gables, FL 33146 USA; 2 Department of Marine Biology and Ecology, Rosenstiel School of Marine and Atmospheric Science, University of Miami, Miami, FL 33149 USA; 3 Viral Information Institute, San Diego State University, San Diego, CA 92182 USA; 4 Computational Science Research Center, San Diego State University, San Diego, CA 92182 USA; 5 Department of Mathematics and Statistics, San Diego State University, San Diego, CA 92182 USA; 6 Institute for Biodiversity and Ecosystem Dynamics, University of Amsterdam, Amsterdam, The Netherlands; 7 Hawaiʻi Institute of Marine Biology, University of Hawai’i at Mānoa, Kāneʻohe, HI 96744 USA; 8 Department of Biology, San Diego State University, San Diego, CA 92182 USA; 9 Botany Department, University of British Columbia, Vancouver, BC V6T 1Z4 Canada; 10 Department of Ecology and Evolutionary Biology, UC Los Angeles, Los Angeles, CA 90095 USA; 11 Scripps Institution of Oceanography, UC San Diego, La Jolla, CA 92037 USA; 12 Red Sea Research Center, King Abdullah University of Science and Technology, Thuwal, 23955-6900 Saudi Arabia; 13 Pacific Islands Fisheries Science Center, National Oceanic & Atmospheric Administration, Honolulu, HI 96818 USA

**Keywords:** Bacteriophages, Coral cover, Phase-shift, Microbialization, Fish biomass, Benthic cover

## Abstract

**Background:**

Predation pressure and herbivory exert cascading effects on coral reef health and stability. However, the extent of these cascading effects can vary considerably across space and time. This variability is likely a result of the complex interactions between coral reefs’ biotic and abiotic dimensions. A major biological component that has been poorly integrated into the reefs' trophic studies is the microbial community, despite its role in coral death and bleaching susceptibility. Viruses that infect bacteria can control microbial densities and may positively affect coral health by controlling microbialization. We hypothesize that viral predation of bacteria has analogous effects to the top-down pressure of macroorganisms on the trophic structure and reef health.

**Results:**

Here, we investigated the relationships between live coral cover and viruses, bacteria, benthic algae, fish biomass, and water chemistry in 110 reefs spanning inhabited and uninhabited islands and atolls across the Pacific Ocean. Statistical learning showed that the abundance of turf algae, viruses, and bacteria, in that order, were the variables best predicting the variance in coral cover. While fish biomass was not a strong predictor of coral cover, the relationship between fish and corals became apparent when analyzed in the context of viral predation: high coral cover (> 50%) occurred on reefs with a combination of high predator fish biomass (sum of sharks and piscivores > 200 g m^−2^) and high virus-to-bacteria ratios (> 10), an indicator of viral predation pressure. However, these relationships were non-linear, with reefs at the higher and lower ends of the coral cover continuum displaying a narrow combination of abiotic and biotic variables, while reefs at intermediate coral cover showed a wider range of parameter combinations.

**Conclusions:**

The results presented here support the hypothesis that viral predation of bacteria is associated with high coral cover and, thus, coral health and stability. We propose that combined predation pressures from fishes and viruses control energy fluxes, inhibiting the detrimental accumulation of ecosystem energy in the microbial food web.

**Supplementary Information:**

The online version contains supplementary material available at 10.1186/s12915-023-01571-9.

## Background

Coral reefs harbor over 30% of the ocean’s biodiversity but are some of the most impacted ecosystems by anthropogenic threats [1]. The complex symbioses and interactions between corals, microbes, and viruses that make up a coral holobiont provide organic matter and nutrient turnover that sustains large trophic webs that extend well beyond the reef [2, 3]. The accretion of corals’ calcium carbonate skeletons generates intricate tridimensional structures that provide habitat, nursery, and breeding grounds for reef and offshore species [4]. The heterogeneous reef structure also fosters niche specialization and biological diversification [5]. In addition to the services provided by corals within the reef ecosystem, coral reefs offer coastline protection from storms and erosion, fisheries, and recreation activities [6, 7]. Despite their unequivocal relevance, the biotic and abiotic factors associated with reef coral cover can be unclear and vary substantially across space and time [8–10]. Any effective efforts to conserve or restore corals and the ecosystem they sustain must consider the reefs’ health status and trajectories across abiotic, biotic, and human scales [11].

The effects of top-down control exerted by large predators on coral reef health (e.g., live coral cover) and the negative impact of their removal by overfishing  represent a paradigm in reef ecology [12–15]. Predation by large fish—such as groupers, snappers, and sharks—controls the abundance, growth rates, and size distributions of smaller fish (herbivores, detritivores, and planktivores) [16, 17]. Predation and overfishing of herbivores are particularly relevant for coral reefs because herbivores control the growth of fleshy algae that can overgrow and kill coral [18–22]. However, the extent of the top-down forces created by predators and their effects on coral reefs may vary significantly [10, 16, 23, 24]: high omnivory, dietary overlaps between species, diet shifts, and external energy inputs may weaken top-down effects resulting in a weak or absent statistical association between fish biomass and coral cover [25, 26]. In some cases, bottom-up effects (e.g., reef structural complexity) impact fish trophic groups more than predation [27]. The combination of bottom-up and top-down forces likely generates feedback loops that may diminish or exacerbate the resulting top-down effects. Therefore, identifying drivers of coral cover requires multi-scale analyses that incorporate multiple biotic, abiotic, and human-driven components of the reef ecosystem.

Microorganisms are critical modulators of coral survival and ecosystem degradation [28–31]. Microbes have high metabolic rates relative to their size, causing large shifts in ecosystem energy allocation even with minor changes in their biomass [32, 33]. However, little is known about the connections and potential synergisms between microbes and the reef’s macro-scale food web components. Heterotrophic bacteria in the benthic boundary layer surrounding a coral surface consume most photosynthetic products fixed by corals and algae [34, 35]. Therefore, benthic primary production connects the microscopic and macroscopic food webs, and changes in energy fluxes at macro-levels affect coral reefs down to microbial scales [36–39]. This is best exemplified in the DDAM (dissolved organic matter, disease, algae, microbes) positive feedback loop [40, 41], where the removal of herbivores facilitates the growth of turf and fleshy macroalgae [38], which produces labile photosynthetic products that, in turn, increase bacterial growth [42, 43]. Uncontrolled bacterial growth fueled by algal exudates consumes oxygen and creates hypoxic zones at the coral-algae interfaces [41, 44, 45], which kills corals, thereby opening benthic space for more algae, sustaining the feedback loop [46, 47]. On reefs where DDAM is most prominent, almost 100% of the ecosystem’s energy has shifted from macroorganisms to the microbial food web, a phenomenon called microbialization [37, 48]. Increased microbial densities in the seawater may also favor the emergence of coral pathogens and decrease resistance to coral bleaching due to dysbiosis in the coral microbiome and its surroundings [49–52]. Despite these connections between microbes and higher trophic levels, very few studies combine both micro and macro data on coral reefs.

Bacteriophages (phages)—viruses that infect and prey on bacteria—represent the most abundant biological entities in coral reefs, but have a poorly understood role in ecosystem structuring [53]. Phage infections can remove almost half of the bacterial biomass from marine surface waters daily via lysis of bacterial cells [54, 55]. Events of elevated lytic activity measured by high viral production and low bacterial abundances are common on coral reefs [56, 57]. These observations lead to the prediction that viral predation of bacteria plays a pivotal role in modulating the speed and magnitude of coral reef microbialization [3, 58]. However, the exact role that phage predation plays in the DDAM positive feedback loop may be multifaceted. High viral predation pressure and bacterial lysis have been predicted to accelerate nutrient transfer from the water column to benthic communities, fueling coral pathogens that cause disease and mortality [58]. However, a negative relationship between virus-to-microbe ratios (i.e., predation pressure) and microbial densities observed on coral reefs across a large geographical scale suggests that in microbialized reefs, viruses exert low predation pressure, which may further increase microbial biomass to the detriment of corals [59–61]. Additionally, phages carry virulence genes that can trigger the emergence of bacterial pathogens [62] when they integrate into their bacterial host genome (lysogeny), establishing a mutualistic relationship with their host [63]. These lysogenic or temperate phages are generally more abundant in microbial-dense reefs and can mediate pathogen invasion of the coral microbiome, ultimately causing tissue necrosis [64, 65] and posing an impending threat to coral reefs.

**Fig. 1:**
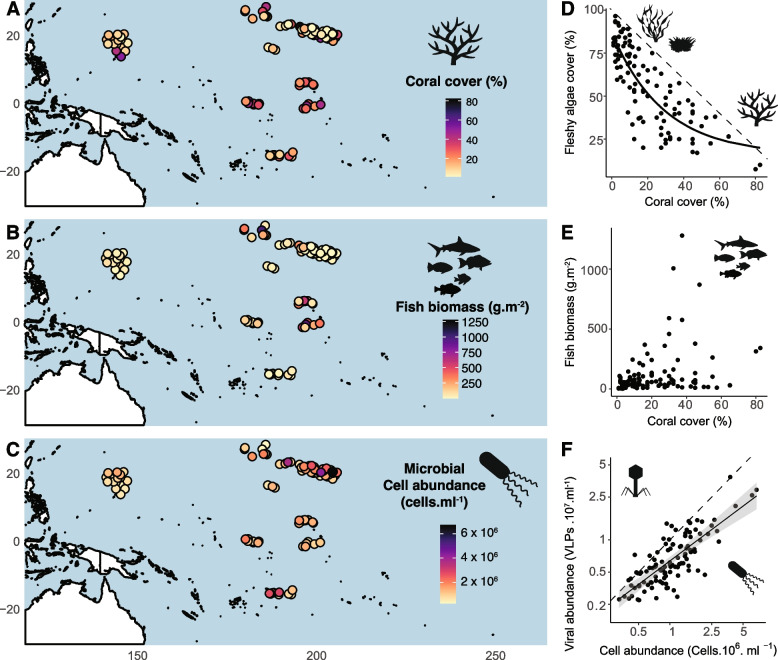
Benthic cover, fish biomass, and microbial abundances at mid-depth (10–15 m) on 110 coral reef communities across the Pacific. **A** Percentage of benthos covered by live scleractinian corals. **B** Fish biomass, as the sum of herbivores, planktivores, invertivores, piscivores, and sharks. **C** Abundance of microbial cells in the water overlying the reef benthos (within 30 cm). **D** Relationship between the percentage of the benthos covered by fleshy algae, as a sum of turf algae and fleshy macroalgae, and the percentage of the benthos covered by scleractinian corals, where the dotted line indicates a proportionally inverse relationship summing up to 100%, and the solid line indicates a non-linear fit. **E** Relationship between total fish biomass and coral cover. **F** Relationship between viral abundances and microbial cell abundances, where the dotted line indicates a 10:1 relationship and the solid line indicates a linear regression in the log–log plot. The benthic, fish, and bacterial data were obtained concurrently at the same sites visited during the NOAA Pacific Reef Assessment and Monitoring Program

To elucidate the role of microscopic predators on coral reef microbialization, we performed an integrative statistical analysis of abiotic, microbial, benthic, fish, and human variables from more than 100 reefs across the Pacific (Fig. 1A, Additional file 1 for the full dataset, and Additional file 2: Table S1 for the summary statistics). We show that turf algae and boundary layer viruses and bacteria have the highest power in predicting coral cover: virus-to-microbe ratios (i.e., high viral predation pressure) positively correlated with coral cover. Our study also indicated that the presence of human populations adjacent to these reefs, which indicates a higher potential for local anthropogenic impacts, alters the relationships between coral cover, microbial densities, viral predation, and fish biomass. Together these findings suggest that viral predation dynamics are the strongest trophic control in the global microbialization of coral reefs.

## Results

Two non-parametric statistical models tested the relationships between biotic and abiotic variables and live scleractinian coral cover: random forests identified the variables of the highest importance in predicting coral cover, and thin-plate splines described the scale of the relationships among these variables. The variables tested here were as follows: the biomass of five dietary groups of fish—herbivores, planktivores, omnivores, piscivores, and sharks (Fig. 1B); the percent cover of turf algae, fleshy macroalgae, and crustose coralline algae (CCA), due to their direct competition with corals for the substrate; [66]; bacterial and viral abundances in the benthic boundary layer due to their potential effects on coral health (Fig. 1C); and dissolved inorganic carbon (DIC) and total alkalinity due to their importance in the coral skeleton accretion and dissolution. These data were collected simultaneously from 110 reef sites in inhabited (*n* = 54) and uninhabited (*n* = 56) islands and atolls in the Hawaiian Archipelago, Marianas Archipelago, and remote Pacific islands. The biotic and abiotic data represent instantaneous measures across an extensive geographical range rather than long-term averages. Preliminary pairwise analyses showed that fleshy algae cover had a negative relationship with coral cover (Fig. 1D, Pearson correlation *r* =  − 0.79), in agreement with previous studies [9]. The total fish biomass, however, did not display a strong relationship with coral cover (Fig. 1E, Pearson *r* = 0.30). The viral abundance was positively correlated with bacterial abundance (Fig. 1F, Pearson *r* = 0.79). All-versus-all pairwise relationships are shown in Additional file 2: Figure S1.

**Fig. 2:**
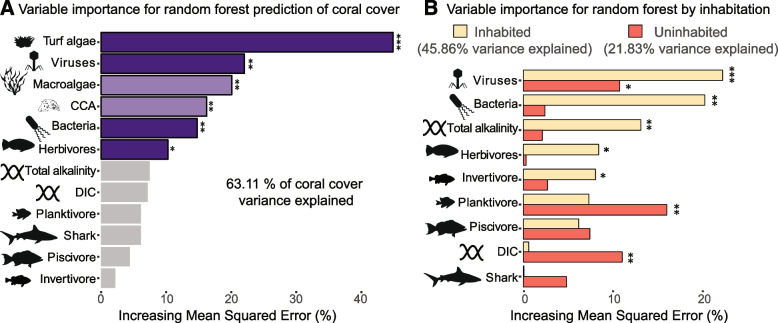
Variable importance from random forests. **A** Variable importance in the random forest model including all benthic, fish, microbial, and water chemistry variables. **B** Variable importance in independent random forests for inhabited (yellow) and uninhabited (orange) sites. In **A**, purple bars indicate variables with *p*-value < 0.05 in the permutation test, while gray bars indicate *p*-values > 0.05. Stars indicate the *p*-values in the random forest permutation test (***p-value < 0.001, **p-value < 0.01, *p-value < 0.05). Light purple indicates variables removed by the conditional random forests

Random forests, a robust non-parametric statistical learning method, indicated that the biological and water chemistry variables investigated here explained 63.11% of the variance in coral cover in the studied reefs (Fig. 2A). The increase in mean squared error (inc.mse) of the random forest model quantified the relative contribution of each variable in predicting coral cover [67]. Benthic algae, viruses, and bacteria were the main contributors to the model prediction power, with turf algae contributing to 44.89% of the random forests’ inc.mse. Water chemistry contributed significantly less to explaining coral cover than biological variables, with dissolved inorganic carbon contributing 7.14% to inc.mse and total alkalinity contributing 7.46% (Fig. 2B). A second random forest model without abiotic variables explained 64.25% of the variance in coral cover (Additional file 2: Figure S2A).

A conditional random forest model tested if covariance between variables interfered with the importance quantification by the random forests. Among the benthic variables tested, turf algae was the only variable listed as highly important in the conditional test, with crustose coralline algae (CCA) and macroalgae dropping in importance (Fig. 2A, light purple bars). Both viruses and bacteria were maintained as high-importance variables and were kept in subsequent analyses using rfPermute, despite their significant positive relationship. To avoid potential confounding effects of substrate cover summing up to 100%, which causes the value of benthic variables to be dependent on each other to some extent, a random forest model where benthic algae were removed was tested. This reduced to 37.56% the variability in coral cover explained by the model, highlighting the well-described importance of turf algae on these reefs [9] (Additional file 2: Figure S2B). Viral abundance was the second most important variable in the model with all variables and the most important variable in the model excluding benthic algae (23.45% inc.mse, *p*-value = 0.0009). Bacterial abundance followed in importance (Additional file 2: Figure S2B; 16.66% inc.mse, *p*-value = 0.003), in addition to the biomass of herbivores, piscivores, and dissolved inorganic carbon (13.60, 12.37, and 11.67% inc.mse, and *p*-values = 0.006, 0.008, and 0.011, respectively). Removing viral and bacterial abundances decreased the model’s explanatory power to 26.78%, indicating a strong relationship between these microbial groups and coral cover. An independent non-parametric test using cubic spline functions in a generalized additive model (GAM) led to similar conclusions as the random forests analysis (Additional file 2: Figure S3, GAM deviance explained = 30.18%). These results demonstrate that turf algae, viruses, macroalgae, CCA, and bacteria are the strongest predictors of coral cover, followed by the biomass of herbivores.

We incorporated the potential effects of local human impacts caused by the populations living in proximity to these reefs (overfishing, pollution, coastal erosion, etc.) in this analysis by testing the effect of inhabitation on the random forest model’s power to predict coral cover [37, 68]. The explanatory power of the model increased to 45.86% in inhabited islands (Fig. 2B). Viral abundance was again the variable with the highest importance (Fig. 2B, 21.28% inc.mse, *p*-value = 0.0009). Bacterial abundance was the second most important variable (18.66% inc.mse, *p*-value = 0.003), followed by alkalinity (15.29% inc.mse, *p*-value = 0.008). To further explore the relationship between local impacts and microbialization, we calculated the bacterial biomass as the product of cell abundances from the present study and the island-specific per-cell biomass quantified previously for a subset of the reefs studied [37]. Bacterial biomass had a negative relationship with the percentage of the benthos covered by calcifying organisms, the sum of coral and crustose coralline algae cover (Additional file 2: Figure S4, linear regression slope =  − 2.6643 and *p*-value = 0.0001). The negative relationship between bacterial biomass and calcifying cover was steeper in inhabited than in uninhabited reefs. However, this relationship was only significant in uninhabited locations (slope =  − 3.595 and *p*-value = 0.07 and slope =  − 1.3361 and *p*-value = 0.0368 on inhabited and uninhabited sites, respectively). The random foress model explained only 21.83% of the variance in coral cover in uninhabited islands. Planktivores, dissolved inorganic carbon, and viruses had the highest importance for the prediction of coral cover on these sites (Fig. 2C).

**Fig. 3:**
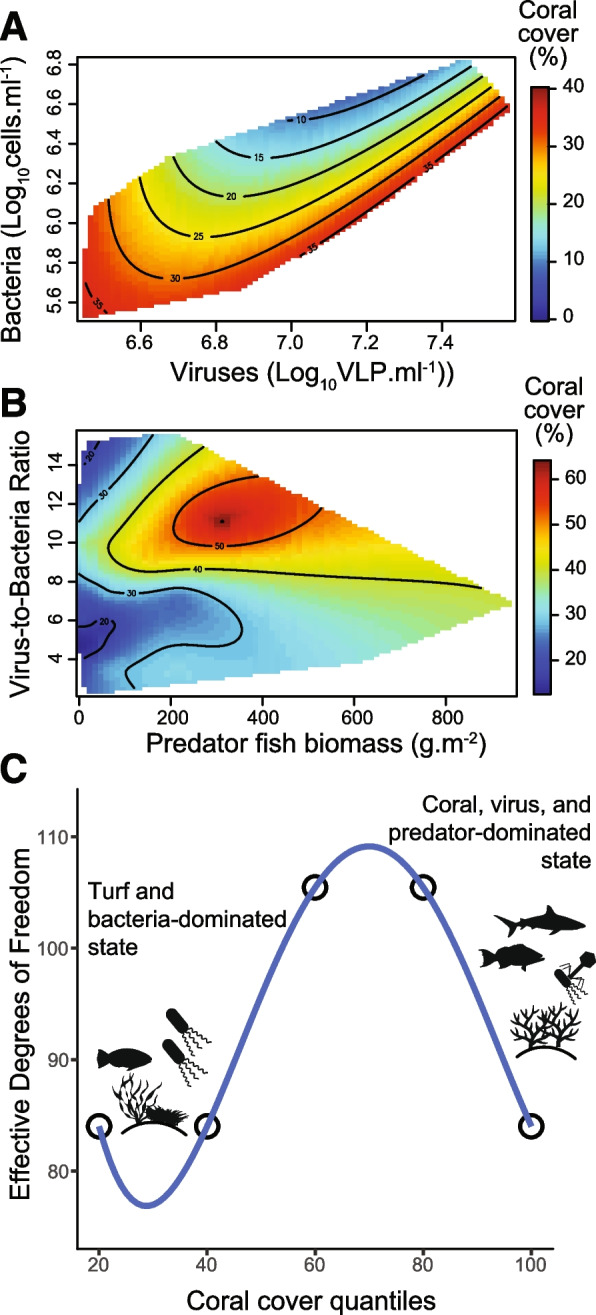
Relationship between microbial and fish predators across the coral cover gradient.** A** Thin plate spline surface prediction with robust smoothing of the relationship between microbial cell abundance, viral abundance, and coral cover. **B** Surface prediction of the relationship between the virus-to-bacteria ratio (VBR), the biomass of predator fish, and coral cover by robust smoothing using a thin plate spline. **C** Effective degrees of freedom across quantiles of coral cover obtained from the cubic smoothing splines

Non-parametric median smoothing spline models revealed the landscape of interactions between the biological variables identified by the random forests across the gradient of coral cover. First, coral cover was investigated as a function of viral and bacterial abundances (Fig. 3A). Corals were predicted to cover 30% or more of the reef substrate where bacterial abundances were low or at higher bacterial abundances when the viral abundances were proportionally higher (high virus-to-bacteria ratio, VBR > 10). In contrast, the increase in bacterial abundance relative to viruses (low VBR) occurred at low coral cover. When including the relationships between microbes, benthos, and fish, the highest coral cover values (> 50%) were predicted in regions of the spline models with a high virus-to-bacteria ratio (VBR > 10) and relatively high predator fish biomass (> 200 g m^−2^) (Fig. 3B). The highest values of predator fish biomass (> 600 g m^−2^) were associated with high VBRs and high coral cover.

The effective degrees of freedom (*edf*) of the smoothing spline models across the coral cover gradient were highest in the middle quantiles of coral cover, increasing from 84 in the lowest and highest quantiles to 105 in the middle quantiles (cover quantiles from 0.2 to 0.8, Fig. 3C). The changes in *edf* indicated that intermediate coral cover reefs have a broader domain in the state variables dimensions compared to reefs at the highest or lowest coral cover values, i.e., more possible combinations of microbial, benthic, and fish parameter values yielded intermediate coral covers compared to the lowest and highest coral cover reefs, where parameter combination ranges were narrower (Figure S2B).

## Discussion

Here we show that viral and microbial densities, in addition to the abundance of benthic algae, are the strongest predictors of coral cover variance in a dataset spanning water chemistry to fish biomass from 110 coral reefs across the Pacific. Mounting evidence indicates that microbial activity causes coral mortality: high respiration rates of dense and fast-growing heterotrophic bacterial communities create hypoxic zones at the interfaces between corals and turf and fleshy macroalgae that release large amounts of labile exudates and can cause coral tissue necrosis [34, 41, 46–48, 69, 70]; terrestrial runoff and nutrient enrichment also fuel microbial overgrowth and hypoxia, sometimes in large dead zones [71]; even large-scale temperature-induced bleaching events may involve bacterial nitrogen metabolism that generates oxidative and nutritional stress in the holobiont [72, 73]. High temperatures also induce virulence in bacterial pathogens [74, 75]. These studies indicate that microbes can be agents of major coral mortality processes. The data presented here add to this body of evidence and suggest that phage predation pressure on bacteria is significantly associated with coral cover, and, presumably, with coral health. Pristine Pacific reefs with high coral cover had high viral predation pressure, indicated by their high virus-to-bacteria ratios (VBR) [60, 61]. We propose that high viral predation pressure may benefit coral reef health and stability by controlling microbialization and the DDAM feedback loop. High lytic viral turnover in healthy reefs could also contribute to nutrient recycling, an additional mechanism explaining Darwin’s Paradox (the presence of high-productivity reef ecosystems in otherwise oligotrophic regions of the Oceans) [58]. Testing this hypothesis will require quantifying the rates of organic and inorganic material released by viral lysis and subsequent effects on coral health and survival.

The high frequency of lysogenic infections on coral reefs with high bacterial densities (above 5 × 10^6^ cells.ml^-1^) was previously shown to decrease VBRs [59]. Lysogeny is a type of infection in which phages integrate into the host chromosome, behaving more like a mutualist than a predator [59]. Lysogeny lowers the VBR by decreasing lytic production relative to the total number of viral infections. Here, we show that the low VBRs in the reef boundary layer (and, drawing from previous studies, lysogeny) are negatively associated with coral cover. The increase in the frequency of lysogenic infections is likely caused by the rise in the total abundance of viral particles coupled with a decrease in diversity observed in degraded reefs [76]. High abundance and low diversity increase the chances of encounters and coinfections by two or more phages, the primary mechanism regulating the establishment of lysogeny [77]. Coinfections typically yield a bimodal distribution of lysogeny across microbial density gradients in marine ecosystems, where lysogeny is favored at low densities in the deep ocean due to slow host growth rates and at high densities in coastal waters due to high encounter rates [76]. High viral abundances have also been associated with increased CRISPR sequences in bacterial communities [78]. The increased CRISPR-mediated resistance against lytic infection may decrease viral particle production; therefore, the two mechanisms may act together to reduce VBR. The weakened phage predation pressure likely promotes microbialization and bacterial-mediated coral death [34, 35, 37, 48].

In our dataset, fish biomass alone showed a poor relationship with live coral cover. While trophic cascades connecting these groups have been extensively described in the coral reef ecology literature, the relationship between fish biomass and coral cover remains unresolved in several regions [17, 22, 39, 79, 80]. Evidence for trophic cascades due to apex predator removal has been weak or absent in the Great Barrier Reef [24, 81]. Previous studies have shown that the tridimensional complexity provided by corals, including dead coral structures, better explains the relationship between corals and fish biomass, suggesting that live coral cover may not represent a proxy for coral reefs’ provision of ecosystem services [82]. However, the presence of live calcifying corals is essential for maintaining the tridimensional complexity over time by counterbalancing the effects of erosion and dissolution [83, 84]. In our dataset, substrate maximum and mean height were not significantly correlated with total fish biomass or coral cover (person correlations *r* < 0.07), suggesting that other biological interactions, rather than substrate complexity, are at play. The association between coral cover and fish biomass became apparent when coral cover was analyzed in the context of fish dietary groups and virus-to-bacteria ratios (Fig. 3B). This indicates that viral and fish predation might act in concert to maintain high coral cover. Previous studies on pristine reefs in the Pacific (most of the same reefs analyzed here) showed that fish biomass distributions take the shape of an inverted pyramid, suggesting that large-bodied predators control reef energy fluxes by constant consumption of herbivores, moving energy up the food web and acting as carbon sinks [17, 80]. The continuous removal of benthic fleshy algae by large herbivores that escape predators by size exclusion tips the balance of the coral-algae benthic competition in favor of corals [9, 85, 86]. Combined with the effects of viral predation of bacteria discussed in the previous paragraph, viral and fish predation together may favor high coral cover on these reefs. Another mechanism contributing to the relationships between corals, fish, and microbes may include the diversity of the coral community, which can modify corals’ contribution to tridimensional complexity and was not analyzed here [84].

Interactions between regional climate patterns, land influences, local inorganic and organic nutrients, and other oceanographic conditions certainly play a role in sustaining coral cover and microbial densities [87–89]. Elevated temperatures, for example, represent one of the most pressing threats to coral survival in the Anthropocene [11, 90]. The low importance of water chemistry in our analyses may have been due to the lack of long-term data. Yet, in the independent analyses of inhabited and uninhabited sites, alkalinity and dissolved inorganic carbon significantly contributed to the prediction of coral cover (Fig. 2C). These results suggest the high importance of these variables in the control calcification in pristine reefs, and that local human impacts disturbing these controls. Local dynamics, such as land inputs, may indirectly affect phage local-scale replication, such as the switch to lysogeny observed during dead zone hypoxic events [91]. This underscores the lack of understanding of the nuanced interactions between chemical oceanography, microbiology, and local impacts [89].

The differences in variable importance in the random forest model between inhabited and uninhabited reefs corroborated previous findings showing that local anthropogenic impacts dramatically increase the proportion of ecosystem energy allocated to microbes versus macroorganisms in the Pacific [37]. Viruses and bacteria were the strongest predictors in inhabited reefs, while planktivores showed the highest importance in uninhabited reefs (Fig. 2C). These fish heavily rely on the reef’s tridimensional complexity [92], and this result could be a simple consequence of a higher coral cover in uninhabited reefs. However, coral cover was not significantly higher on these reefs (t-test *p* = 0.07436). Alternatively, the importance of planktivores may result from their reliance on the larval supply offered by cryptobenthic fish, which have a disproportionate contribution to the supply of planktonic larvae relative to their standing stock biomass [93]. Their constant transfer of biomass and energy to planktivores may significantly contribute to keeping the ecosystem’s energy from the microbial food web.

Reefs with intermediate coral cover values displayed higher degrees of freedom in the spline models describing the relationships between fish, microbial and benthic variables compared to coral- or algae-dominated reefs (Fig. 3C). The splines suggest that a more defined combination of values for fish and microbial variables characterize the extremely low or high coral cover reefs compared to reefs at intermediate states. This observation is consistent with the idea that reefs with intermediate coral cover represent alternative unstable transition states between coral and algae dominance [9, 94]. Our results broaden this concept by introducing the viral communities to the landscape of biotic relationships describing the transition from coral to algae dominance [3].

## Conclusion

**Fig. 4:**
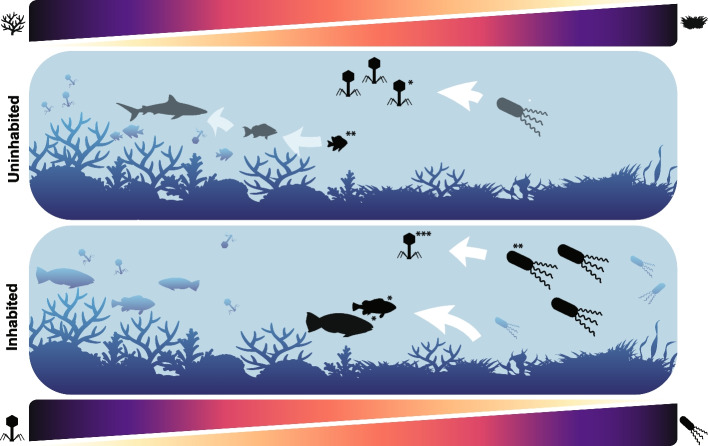
Conceptual figure illustrating the relationship between predation pressure by viruses, fish predators, and coral cover. Each panel indicates the reef components with the highest importance in uninhabited (top) and inhabited (bottom) reefs according to the statistical learning approach, in addition to their relationships. Asterisks indicate the significance of each variable in the Random Forest model (***p-value < 0.001, **p-value < 0.01, *p-value < 0.05). Abiotic variables (DIC and alkalinity) were omitted from the figure for simplicity. The phage icon indicates viral predation pressure, not abundance. For a legend of the fish icons, please see Fig. 2

Drawing from the mechanistic links proposed by previous studies focused on each component of the reef food web [17, 48, 59, 80], we propose that the predation pressure from both viral and fish predators act in combination to control the reef's energetic fluxes and maintain reef health (Fig. 4). If viral and fish predators operate in concert to favor high coral cover, their interactions are expected to generate feedback loops in response to external disturbances: herbivore removal lifts the pressure on algae and affects reefs down to microbial scales [35, 38, 39, 86]; since large changes in ecosystem energy allocation result from small changes in microbial biomass, synergistic effects between viral and fish predation are predicted to arise [32]. This amplification effect may set thresholds and the speed of changes upon disturbance [3, 11]. Identifying potential synergic (or additive) effects between these groups will move us toward accurately tracking and predicting coral reefs’ trajectories.

## Methods

The dataset analyzed here was generated by the Rapid Ecological Assessment (REA) protocol as part of the Pacific Reef Assessment and Monitoring Program (Pacific RAMP) of the Coral Reef Ecosystem Program (CREP), National Oceanic and Atmospheric Association (NOAA). The data was collected during the RAMP cruises of 2012, 2013, and 2014. Data from the mid-depth strata (10–15-m depth) was analyzed [95].

### Abiotic variables

Oceanographic data was retrieved from the National Coral Reef Monitoring Program: Water chemistry of the coral reefs in the Pacific Ocean Dataset by the Ecosystem Sciences Division, Pacific Islands Fisheries Science Center, accessed on March 4, 2020 [96]. Briefly, water was collected between 8- and 12-m depth using a diver-operated 2L Niskin bottle. Immediately after the dive, the sample was transferred to a 500-ml glass flask, and 200 μl of saturated mercuric chloride was added to each bottle. The sample was sealed with stoppers using grease and preserved until laboratory processing. In the laboratory, DIC was analyzed coulometrically [97]. Total alkalinity was measured using the potentiometric titration method [98]. Temperature, salinity, and conductivity were measured in situ using conductivity, temperature, and depth (CTD) sensors. These abiotic data do not represent long-term averages, but rather instantaneous measures taken at the time of sampling for benthic cover, microbial abundance, and fish biomass.

### Benthic community composition

A one-stage stratified random sampling design was employed, and sites were randomly selected around each island/atoll. Surveys at each site were conducted within two 18-m belt transects. Photographs were taken every 1 m from the 1 m to the 15-m mark with a high-resolution digital camera mounted on a pole. This work generated 30 photographs per site, which were later analyzed using the computer program CoralNet.

### Fish biomass

Divers conducted fish surveys using the stationary-point-count (SPC) method at preselected REA sites in the forereef habitat strata. The REA site surveys were performed using a 30-m transect line set along a single depth contour. A team of two divers conducted two adjacent and simultaneous SPC surveys. Once a transect line was deployed, the two divers moved to the 7.5-m and 22.5-m marks on this transect line to start their SPC surveys. Each of these marks or points, with one diver at each, served as the center of a visually estimated cylindrical survey area with a radius of 7.5 m. During the first 5 min, divers created a list of all fish species within their cylinder. Afterward, divers went down their respective species lists, created from their work during the initial 5 min of a survey, sizing and counting all individuals within their cylinder, one species at a time. Cryptic species missed during the initial 5 min of a survey could still be counted, sized, and added to the original species list.

### Microbial data

Microbial and viral abundance data were collected from the same sites as benthic and fish data according to previously described methods [99]. Briefly, water samples (2 l) were collected utilizing Hatay-Niskin bottles at 30 cm above the benthos (8–12-m depth). Samples were collected between 10:00 and 12:00 local time and processed on the ship within 4 h of collection. Water subsamples (1 ml) were fixed with paraformaldehyde (2% final concentration), stained with SYBR Gold, filtered through a 0.02-μm Anodisc filter (Whatman), stained with SYBR Gold, and enumerated by epifluorescence microscopy. Viral and microbial abundances were previously published [59]. Briefly, particles smaller than 0.2 μm were defined as viral-like particles (VLPs), and particles between 0.2 and 2 μm were defined as bacteria. At least ten fields of view (FOV) were quantified per sample. The total number of VLPs and microbial cells per sample was calculated by multiplying the average number of particles per FOV by the number of FOVs per filter where a 1-ml seawater sample was filtered. Microbial biomass was calculated for a subset of sites for which mean microbial cell volume at the island site was available from a previous study [37]. Microbial cell volumes (μm^3^) were converted to mass in wet weight (g) using size-dependent relationships for marine microbial communities [100]. Cell volume was converted to dry weight using a linear relationship [100].

### Statistical learning analysis

The variables analyzed in the combined dataset were as follows: viral abundance (Log_10_ of viral-like particles (VLP) per ml of seawater), microbial cell abundance (Log_10_ of microbial cells per ml of seawater), hard coral cover (%), turf algae cover (%), fleshy macroalgae cover (%), crustose coralline algae (CCA) cover (%), herbivore fish biomass (in g m^−2^), invertivore fish biomass (g m^−2^), piscivore fish biomass (g m^−2^), planktivore fish biomass (g m^−2^), and shark biomass (g m^−2^), dissolved inorganic carbon (μmol kg^−1^), and total alkalinity (μmol kg^−1^), for a total of 13 variables [95]. First, a permutational supervised regression random forest was applied to the entire dataset using hard coral cover as the predicted variable. Random forests were performed using the R package rfPermute [101, 102]. A conditional random forest was used in the R package party to account for the potential covariance between variables and examine whether it interfered with the importance analysis by the random forest. Among the benthic variables, turf algae was the only variable listed as highly important, with CCA and macroalgae dropping in importance compared to the microbial variables ranked as highly important in the rfPermute. Both viruses and bacteria were maintained as high-importance variables and were kept in subsequent analyses using rfPermute. The dataset was split into sites around inhabited (*n* = 55) and uninhabited islands (*n* = 56) to test the potential effects of local anthropogenic impact. A permutational supervised regression random forest was applied independently to the two datasets. A total of 1000 trees with 1000 permutations were grown in all the random forests. Important variables for predicting hard coral cover were selected based on their contribution to the random forest mean squared error and *p*-value < 0.05 in the permutation test. The mean squared error diagnostic plot showed that the error settled, indicating that enough trees were built.

### Generalized additive models with cubic splines

The semi-parametric generalized additive model (GAM) was built using cubic regression splines as a smoothing term, defined by a modest-sized set of knots spread evenly through the covariate values [103]. They are penalized by the conventional integrated square second derivative cubic spline penalty. The data was modeled in the package mgvc in R [102, 103]. The relative importance of variables was tested using the relaimpo package in R by calculating the relative contribution to the *R*^2^ of the linear model of coral cover and the predicted cubic splines in GAM [104]. The *R*^2^ partition is performed by averaging over orders [105] with bootstrap (samples = 1000). The relative importance metrics tested were as follows: LGM, which is the *R*^2^ contribution averaged over orderings among regressors; Last, which measures variable contribution when included last; First, which measures variable contribution when included first, representing the squared covariance between *y* and the variable; and Pratt, which is the product of the standardized coefficient and the correlation.

### Thin plate splines

The variables showing the highest importance from the random forests were modeled using the smoothing thin-plate spline in the R package fields [102, 106]. These splines solve a minimization problem that fits piecewise cubic polynomials with continuous first and second derivatives. The median cubic smoothing spline is the robust version of the traditional cubic smoothing spline. We analyzed the smoothing spline parameter obtained by generalized cross-validation (GCV) based on the quantile criterion to explore the variable relationships across the coral cover gradient. The effective degrees of freedom (edf) for quantiles of coral cover in the dataset were obtained with the estimated quantiles varying from 0.2 to 0.8 using the standard thin plate spline and an algorithm based on pseudo data to compute robust smoothers based on a weight function [106, 107]. Edf indicates the variability of the data, defined as the degree of the equivalent polynomial fit needed to generate the median and the quantile smoothing splines.

## Supplementary Information

**Additional file 1. **Comma-separated file containing site-level data for the 110 reef sites analyzed in this study. Data includes location (geographic region, island, site name, geographical coordinates), date of sampling, type of reef, depth, benthic cover (hard coral, crustose coralline algae, sum of calcifying organisms, macroalgae, turf algae, sum of fleshy organisms, and sand), viral and bacterial abundance, virus-to-microbe ratio, fish biomass (herbivore, invertivore, planktivores, piscivores, sharks, and others), dissolved inorganic carbon, and total alkalinity.

**Additional file 2: Table S1.** Summary statistics of variables used in statistical learning analyses and splines. **Figure S1.** Pairwise relationships between all variables analyzed in this study. **Figure S2.** Variable importance plots for random forests. **Figure S3.** Variable importance in the generalized additive model (GAM) using cubic spline smoothing. **Figure S4.** Relationship between microbial biomass and benthic cover of calcifying organisms.

## Data Availability

All data analyzed in this manuscript are available as Additional file 1 in comma-delimited format. All datasets and R codes for statistical analyses and visualization are available through the FigShare repository under the project “Viral predation pressure on coral reefs” (https://doi.org/10.6084/m9.figshare.22255564.v1 [95] and https://doi.org/10.6084/m9.figshare.22255573.v1 [102]).

## Abbreviations

CCA: Crustose coralline algae
CREP: Coral Reef Ecosystem Program
CTD: Conductivity, temperature, depth
DDAM: Dissolved organic matter, disease, algae, microbes
DIC: Dissolved inorganic carbon
*edf*: Effective degrees of freedom
FOV: Fields of view
GAM: Generalized additive model
GCV: Generalized cross-validation
NOAA: National Oceanic and Atmospheric Administration
RAMP: Reef Assessment and Monitoring Program
REA: Rapid Ecological Assessment
SPC: Stationary-point-count
VBR: Virus-to-bacteria ratio
