# Supplementary material for: Viral predation pressure on coral reefs
Source: BMC Biol. 2023 Apr 11;21:77. doi: 10.1186/s12915-023-01571-9 (PMC10088212; doi:10.1186/s12915-023-01571-9)
Supplement: Supplementary file 2 — Additional file 2: Table S1. Summary statistics of variables used in statistical learning analyses and splines. Figure S1. Pairwise relationships between all variables analyzed in this study. Figure S2. Variable importance plots for random forests. Figure S3. Variable importance in the generalized additive model (GAM) using cubic spline smoothing. Figure S4. Relationship between microbial biomass and benthic cover of calcifying organisms. [file 12915_2023_1571_MOESM2_ESM.docx]

**Supplementary Figures and Tables**

**Table S1.** Summary statistics of variables used in statistical learning analyses and splines.

|  | Hard Coral (%) | CCA (%) | MA (%) | Turf Algae (%) | Viruses (Log10 VLP/ml) | Cells (Log10 Cells/ml) | Herb. | Invert. | Plankt. | Pisciv. | Sharks | DIC | Total Alkalinity |
| --- | --- | --- | --- | --- | --- | --- | --- | --- | --- | --- | --- | --- | --- |
| Min. | 1 | 0 | 0 | 0 | 6.44 | 5.52 | 0.03 | 0.47 | 0.01 | 0 | 0 | 1930 | 2218 |
| 1st Qu. | 6.5 | 2.5 | 1 | 26.12 | 6.65 | 5.85 | 7.94 | 4.21 | 1.32 | 2.32 | 0 | 1979 | 2291 |
| Median | 15 | 7 | 6 | 44.25 | 6.77 | 6 | 16.22 | 7.35 | 5.30 | 11.68 | 0 | 1997 | 2308 |
| Mean | 21.21 | 13.1 | 10.48 | 45.38 | 6.82 | 6.01 | 20.20 | 12.62 | 15.43 | 37.05 | 21.39 | 1993 | 2302 |
| 3rd Qu. | 32.25 | 22.5 | 14.75 | 66.5 | 6.96 | 6.17 | 25.04 | 14.21 | 18.14 | 35.27 | 0 | 2004 | 2317 |
| Max. | 82.5 | 62.5 | 70 | 96 | 7.59 | 6.83 | 86.94 | 145.28 | 252.42 | 811.94 | 515.05 | 2063 | 2336 |

**
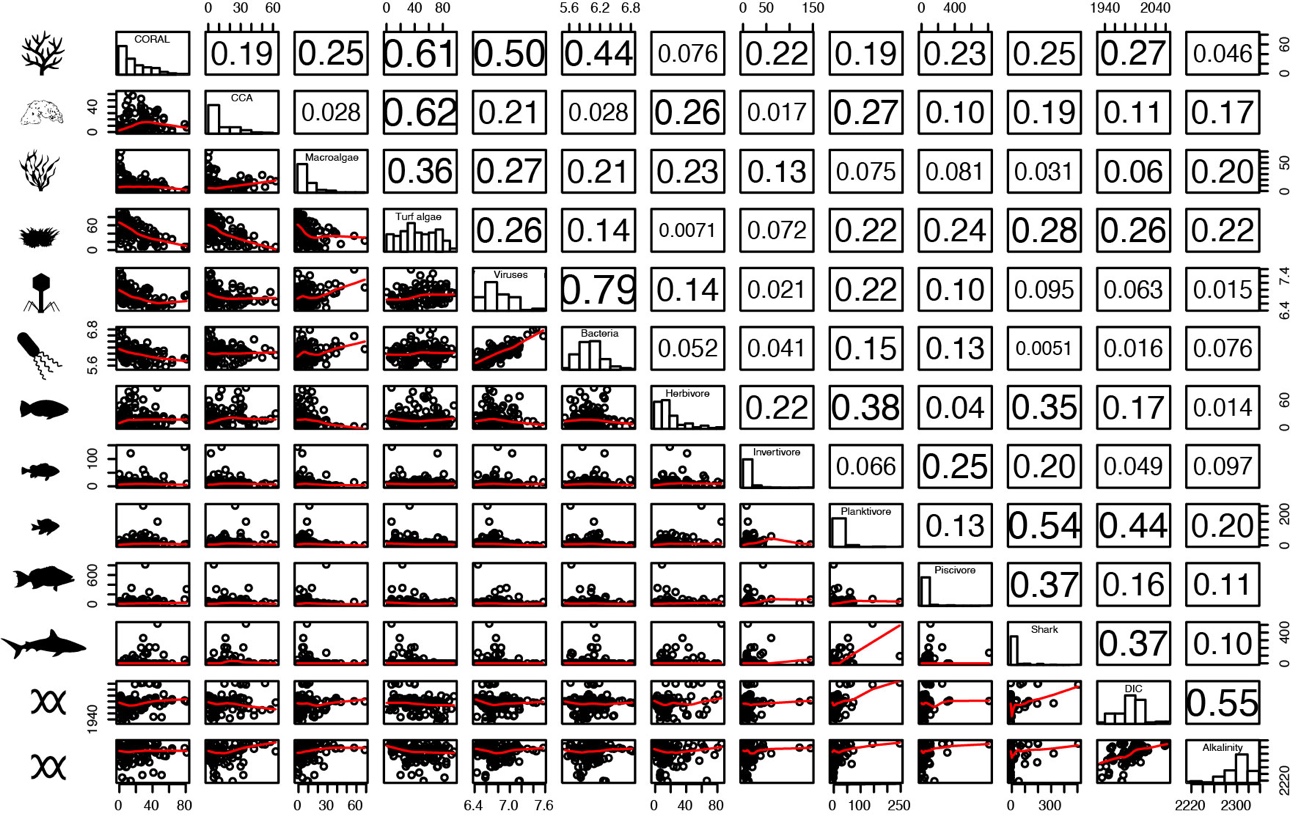
**

**Figure S1.** Pairwise relationships between all variables analyzed in this study. The red lines indicate a locally weighted scatterplot smoothing line. The histogram shows the frequency distribution of each single variable. The numbers on the top-right side of the matrix indicate pairwise correlation tests between variables.

**A**

**
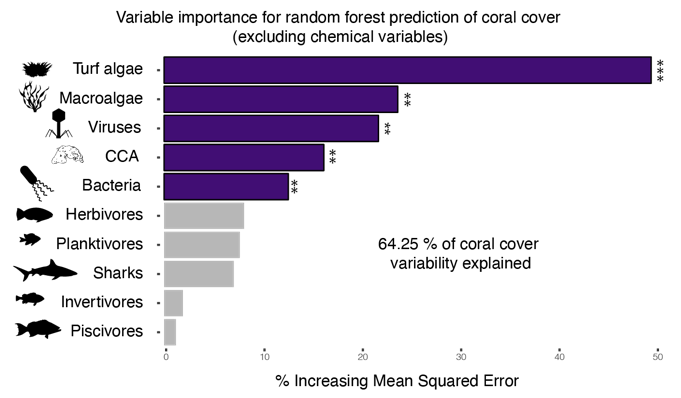
**

**B**

**
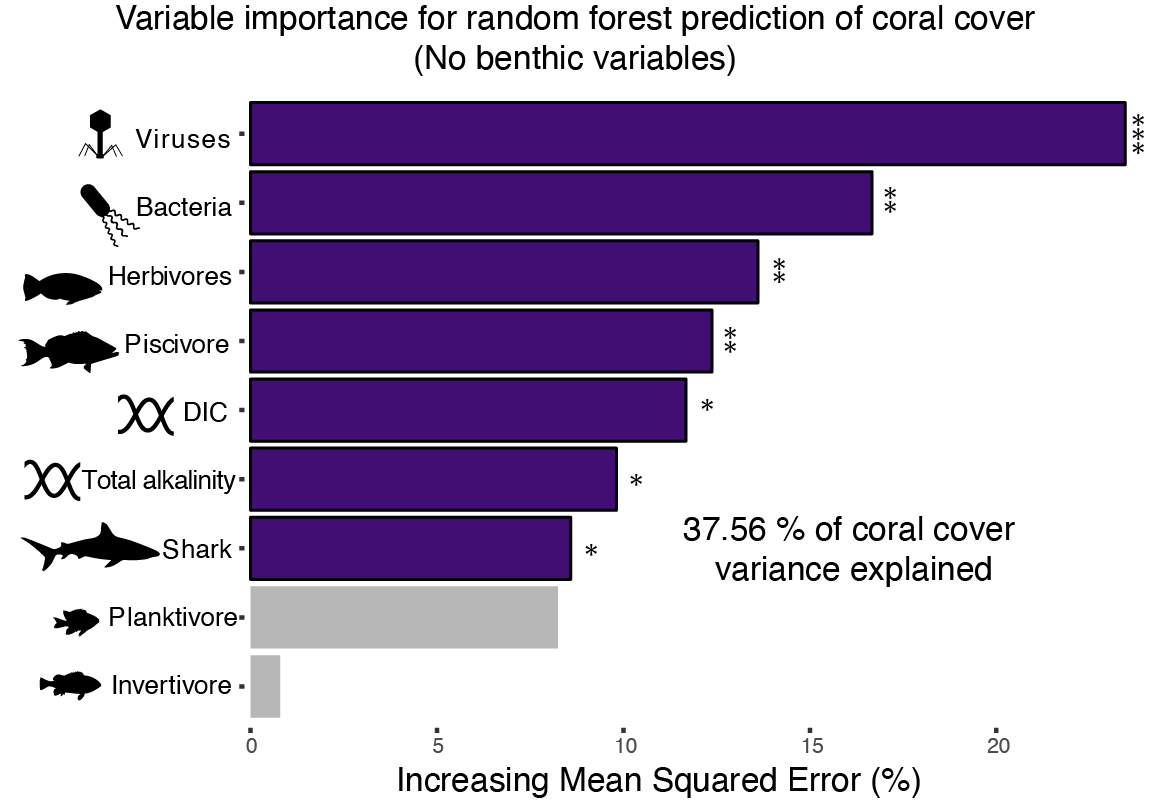
**

**Figure S2.** Variable importance plots for random forests. **A)** Variable importance plot from the random forest model with benthic, fish, and microbial variables only (no water chemistry). **B)** Variable importance plot from the random forest model with fish, microbial, and chemical variables only (no benthic cover). Purple bars indicate variables with p-value < 0.05 in the permutation test, while grey bars indicate p-values > 0.05. Stars indicate the p-values in the random forest permutation test (*** = p-value < 0.001, ** = p-value < 0.01, * = p-value < 0.05).

**Figure S3.** Variable importance in the generalized additive model (GAM) using cubic spline smoothing. Importance was measured with four different methods: LGM, which is the R^2^ contribution averaged over orderings among regressors; Last, which measures variable contribution when included last; First, which measures variable contribution when included first, representing the squared covariance between y and the variable; and Pratt, which is the product of the standardized coefficient and the correlation.

**Figure S4.** Relationship between microbial biomass and benthic cover of calcifying organisms. The grey circles indicate inhabited sites (n = 23), and the black triangles indicate uninhabited sites (n = 47). The analyses include a subset of the sites analyzed in this study, for which microbial cell size was available and allowed to calculate microbial biomass. The linear regression for the whole dataset has a slope of -2.66 and p-value = 0.0001. The linear regression for inhabited sites has a slope of -3.59 and p-value = 0.0759, and in uninhabited sites, a slope of -1.33 and p-value = 0.0368.
